# Food for thought – ILC metabolism in the context of helminth infections

**DOI:** 10.1038/s41385-022-00559-y

**Published:** 2022-08-31

**Authors:** Marcel Michla, Christoph Wilhelm

**Affiliations:** Unit for Immunopathology, Department of Clinical Chemistry and Clinical Pharmacology, University Hospital Bonn, University of Bonn, 53127 Bonn, Germany

## Abstract

Helminths are multicellular ancient organisms residing as parasites at mucosal surfaces of their host. Through adaptation and co-evolution with their hosts, helminths have been able to develop tolerance mechanisms to limit inflammation and avoid expulsion. The study of helminth infections as an integral part of tissue immunology allowed us to understand fundamental aspects of mucosal and barrier immunology, which led to the discovery of a new group of tissue-resident immune cells, innate lymphoid cells (ILC), over a decade ago. Here, we review the intricate interplay between helminth infections and type 2 ILC (ILC2) biology, discuss the host metabolic adaptation to helminth infections and the metabolic pathways fueling ILC2 responses. We hypothesize that nutrient competition between host and helminths may have prevented chronic inflammation in the past and argue that a detailed understanding of the metabolic restraints imposed by helminth infections may offer new therapeutic avenues in the future.

## Introduction

Parasitic helminths are large multicellular organisms residing at mucosal and non-mucosal tissues of their host. As ancient pathogens, the evolution of helminths is intimately intertwined with human evolution and our ancestors suffered from chronic and recurrent helminth infections^1^. Rarely seen in Western, industrialized countries nowadays, chronic helminth infections are still endemic in developing countries. However, despite creating tissue damage by migrating through or residing in tissues of the host organism including the muscle, intestine, lung and skin to complete their life cycle, in most cases helminth infections do not result in persistent and uncontrolled inflammation in a well-nourished population^2–5^. However, helminth infections can cause severe problems such as stunting in undernourished children^6^. Lack of overt immune activation and inflammation has been largely attributed to the intimate evolutionary relationship between the host and parasitic helminths, as worms have developed mechanisms to induce tolerance, tissue maintenance and repair^1,7^. This specific host-pathogen interaction allows the usage of helminth infections to study not only basic concepts of protective tissue immunology but also mechanisms of immune tolerance, tissue maintenance and restoration. In general, infections with tapeworms, flukes or roundworms induce innate and adaptive type 2 immune responses^8^. While innate type 2 immune responses include the induction of tissue-resident alternatively activated macrophages (AAM), activation of mast cells and eosinophils, the adaptive immune response mainly comprises the generation of adaptive T helper 2 (Th2) cells^9^. Furthermore, the study of helminth immunology was pivotal in the discovery of a new innate type 2 cell population, type 2 innate lymphoid cells (ILC2) over a decade ago. In this review, we discuss the aspects of basic mucosal immunology that studies using parasitic helminths have brought to the community by focusing on how helminth infections shaped our current understanding of ILC2 biology, metabolism and function.

## The discovery of ILC2

To study helminth infections in vivo, mouse models of intestinal helminth infection are mainly based on three model parasites, *Nippostrongylus brasiliensis*, *Heligmosomoides polygyrus bakeri* and *Trichuris muris*, with the gastrointestinal rat nematode *N. brasiliensis* probably being the most frequently used for studies of mucosal immunology. Both *N. brasiliensis* and *H. polygyrus* infect the small intestine of mice^10–12^, while *T. muris* is found in the caecum and proximal colon^13^. *N. brasiliensis* induces a strong type 2 immune response and is rapidly expelled, making it an excellent model for studying protective type 2 immune responses to helminths in mice. In contrast, the natural murine parasite *H. polygyrus* establishes a chronic infection in C57BL/6 mice in contrast to more resistant BALB/c mice^14–16^. *T. muris*, depending on the infectious dose, can generate either chronic persistent infections, characterized by a Th1 response and the production of the cytokine interferon (IFN)-γ (low dose infection with ∼25 eggs), or acute infections cleared by a strong Th2 response with the production of interleukin (IL)-5, IL-9, and IL-13 in response to high amounts of eggs (∼150 eggs). This dose-dependent switch in the immune response provides an excellent model to study protective anti-helminth immunity^17–19^.

Before the discovery of ILC2, the production of the type 2 cytokines IL-4, IL-5, IL-9, and IL-13 was mainly attributed to Th2 cells^20^. However, it became clear several years before the first formal classification and identification of ILC2 as a distinct and separate subset, that a population of innate immune cells must exist^21^. This initial observation eventually led to the discovery and first characterization of ILC2 as a separate immune cell subset in the mesenteric adipose tissue^21^ or in the intestine in the context of infections with *N. brasiliensis*^22,23^ and *T. muris*^24^. In general, ILC have been classified into three distinct subsets, ILC1, ILC2, and ILC3 resembling the discrete T helper cell subsets Th1, Th2 or Th17 cells based on the expression of characteristic transcription factors and signature cytokines^25^. ILC1 including natural killer (NK) cells express T-bet (encoded by *Tbx21*) and produce the effector cytokine IFN-γ. ILC1 are implicated in protecting against intracellular pathogens such as *Toxoplasma gondii* but have also been connected to the pathophysiology of inflammatory bowel disease (IBD)^26–28^. ILC2 express the transcription factor GATA3 and produce the cytokines IL-4, IL-5, IL-9, IL-13, and amphiregulin, mediating anti-helminth immunity^13,22,23,29^. Besides this protective function, chronic activation and dysregulation of ILC2 may also result in ILC2-driven pathology in the context of allergies and asthma^28,30^. In contrast, ILC3 are characterized by the expression of the transcription factor RORγt and the cytokine IL-22, mediating anti-bacterial responses^28,31–33^, but also chronic inflammatory conditions such as psoriasis or IBD^34–36^.

## The protective function of ILC2 in helminth infections

**Fig. 1:**
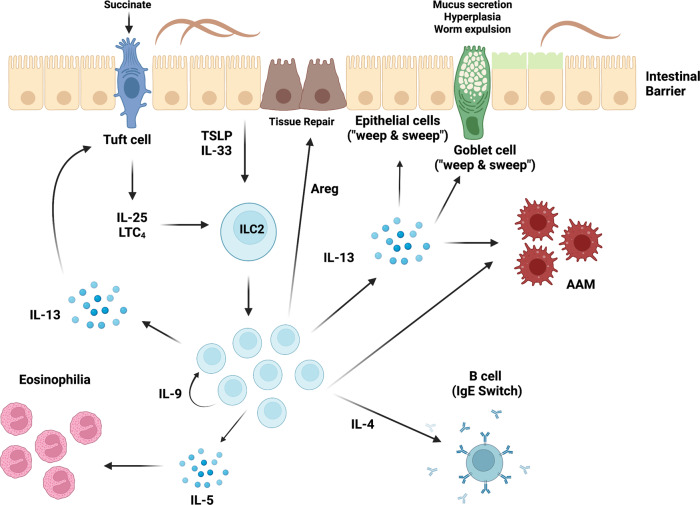
ILC2-mediated anti-helminth immunity. Upon intestinal helminth infection, mucosal epithelial barrier sites release the alarmins IL-33 and TSLP, while chemosensory tuft cells are activated by succinate to secrete IL-25 and the leukotriene LTC4 activating ILC2. ILC2 release IL-13 acting on alternatively activated macrophages (AAM) and promoting epithelial, goblet and tuft cell hyperplasia resulting in the “weep and sweep” response and expulsion of intestinal helminths. The ILC2-derived cytokines amphiregulin (Areg) and IL-9 mediate tissue repair or autocrine survival and maintenance of ILC2, respectively. IL-13 further potentiates IL-25 release from tuft cells and IL-4 induces a class switch to IgE producing B cells, while IL-5 triggers eosinophilia.

The protective role of ILC2 upon helminth infection has been studied extensively and different mechanisms of ILC2 biology promote and contribute to anti-helminth immunity. In particular, ILC2-derived IL-13 was shown to be essential for protective anti-helminth responses by directly acting on intestinal epithelial and goblet cells, inducing hyperplasia, excessive mucus production and increased muscle contractility, culminating in a “weep and sweep” response assisting worm expulsion^37,38^ (Fig. 1). In addition to limiting parasite burden, ILC2-derived IL-4 induces class-switching to IgE-producing B cells^39,40^, while IL-13 and amphiregulin suppress inflammation, promote tissue repair and induce wound healing by acting on epithelial cells and alternatively activated macrophages (AAM)^41^ (Fig. 1). The maintenance of ILC2 responses is critically dependent on the autocrine survival factor IL-9^29,42^ (Fig. 1), manifesting in aggravated damage and impaired restoration of lung function of IL-9R-deficient mice in response to infections with larval stages of *N. brasiliensis* traveling through the lung before entering the intestine^29^. Although administration of neutralizing IL-9 antibodies has been shown to impair *T. muris* and *H. polygyrus bakeri* expulsion from the intestine^43–46^, the direct effect of ILC2-derived IL-9 in this context remains to be revealed.

## Activation of ILC2 by helminths

In general, tissue damage created by intestinal infection with helminths triggers the epithelium to produce the alarmin cytokines IL-33, IL-25 and TSLP resulting in the activation of ILC2. Moreover, besides epithelial cells, stromal cells are known as potent producers of IL-33^47–51^. Mice lacking IL-25 or IL-33 show delayed expulsion of *N. brasiliensis*, *T. muris*, *T. spiralis* and *H. polygyrus*^48,52–56^ and IL-25-deficient BALB/c mice are completely unable to expel adult *H. polygyrus*^57^, corroborating the importance of these cytokines for initiating a protective anti-helminth immune response. Furthermore, combined IL-25- and IL-33-deficiency completely ablates the expansion of ILC2 consequently leading to severe defects in *N. brasiliensis* expulsion^23^. Vice versa, protective anti-helminth immunity can be induced by delivery of recombinant IL-25 to Rag-deficient mice leading to the activation of ILC2 and enhanced production of the effector cytokines IL-5 and IL-13^22,57^. Other than IL-33 and IL-25, TSLP is involved in the protective function only against *T. muris*, but not *N. brasiliensis* or *H. polygyrus* infections, as TSLP is mainly expressed in the large intestine^58,59^. Interestingly, only if applied at an early time point after infection with *T. muris*, exogenous IL-33 administration can promote worm expulsion^56^, suggesting that early activation of ILC2 may be essential for efficient worm expulsion.

Apart from facilitating the discovery of ILC2, helminth infections proved elemental in the recognition of tuft cells, a rare chemosensory cell type in the intestinal epithelium, as essential initiators of type 2 immunity. Tuft cells provide the long-sought essential source of intestinal IL-25, required for the activation of ILC2 and lack of tuft cells results in abrogated hyperplasia, compromised worm expulsion and defective mucosal type 2 immunity^60^. Secretion of IL-4 and IL-13 from ILC2 upon exposure to IL-25 is not only inducing goblet cell activation and hyperplasia, but also employs a feed-forward loop by promoting tuft cell differentiation and hyperplasia and thus further production of IL-25^61,62^. However, this tuft cell-ILC2 axis is mainly described for the murine small intestine, although certain nematodes (*T. muris*) mainly reside in the caecum or distal colon. Sensing the of the tricarboxylic acid (TCA) cycle intermediate succinate can also activate tuft cells. Succinate binds to the succinate receptor 1 (SUCNR1) initiating a signaling cascade that results in the release of IL-25 in a TRPM5-dependent manner^63–65^. This activation loop appears to be essential for the induction of type 2 immunity in the context of the protist *Tritrichomonas*, but neglectable for the induction of anti-helminth immunity.

Remarkably, helminths possess mechanisms able to interfere with the activation of ILC2 even in peripheral organs such as the lung^66^ through the release of excretory-secretory (ES) products. The *H. polygyrus*-derived protein HpARI (*H. polygyrus* Alarmin Release Inhibitor) sequesters IL-33 within the nucleus of necrotic cells, preventing its release and thus ILC2 activation^67^. As a result, delivery of HpARI directly to mice potently inhibited ILC2-mediated allergen responses to *Alternaria Alternata* and impaired the expulsion of *N. brasiliensis*^67^. This concept was then expanded to another ES-protein, *H. polygyrus* Binds Alarmin Receptor and Inhibits (HpBARI)^68^. HpBARI binds to the ST2/IL-33 receptor complex preventing IL-33 signaling and thus the induction of allergen-induced airway inflammation^68^. These findings highlight the propensity that helminths possess to directly modulate immune responses to prevent expulsion.

## Neuro-immune control of helminth infections

**Fig. 2:**
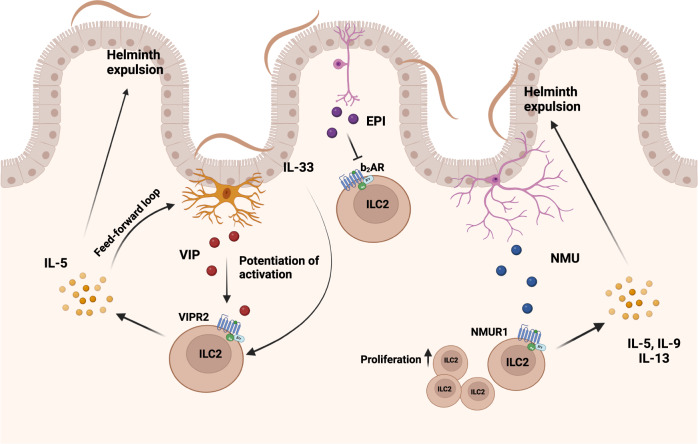
Nervous activation of intestinal ILC2 during helminth infection. Intestinal sensory neurons release vasoactive intestinal peptide (VIP), neuropeptide neuromedin U (NMU) or epinephrine (EPI). Feeding-induced VIP potentiates (IL-33-mediated) activation of ILC2, resulting in increased production of IL-5 and accelerated helminth expulsion. In addition, secreted IL-5 induced a feed-forward loop by acting on VIP-producing neurons to further boost the release of VIP. Helminth infections activate cholinergic neurons to produce neuromedin U enabling rapid ILC2 proliferation and release of IL-5, IL-9, and IL-13 enhancing helminth expulsion. Adrenergic neurons release EPI inhibiting ILC2 function, impairing ILC2-mediated anti-helminth immunity.

Besides the release of IL-25, the latest data also suggest an active role of tuft cells in the production of cysteinyl leukotriene LTC_4_, a known activator of ILC2 resulting in the release of IL-5 and IL-13 to support anti-helminth defense^62^. The neurotransmitter acetylcholine (ACh) can also be directly synthesized by ILC2 and genetic disruption of ACh synthesis in ILC2 results in reduced immunity to *N. brasiliensis* infection^69^. Furthermore, intestinal ILC2 colocalize with cholinergic neurons expressing neuropeptide neuromedin U (NMU) and enteric neurons were shown to release NMU in response to helminth products^70^. ILC2 express the NMU receptor 1 and stimulation with NMU induces rapid proliferation, activation and production of the effector cytokines IL-5, IL-9, and IL-13^70,71^. Exogenous supplementation of NMU during *N. brasiliensis* infection led to accelerated worm expulsion, while NMU-deficient mice displayed increased worm burden and a reduction in ILC2-mediated allergic airway inflammation^71^. This further emphasizes the essential role of neuronal activation of ILC2 in helminth clearance (Fig. 2). Besides NMU, vasoactive intestinal peptide (VIP) released from neurons in response to feeding, is an important modulator of ILC function. VIP signals through the VIP receptor 2 (VIPR2) on ILC2 and promotes the production of IL-5 and thus the recruitment of eosinophils^72^. Additionally, VIP acts in concert with IL-33 to activate mammalian target of rapamycin (mTOR), which increases glycolysis, potentiates the production of intestinal ILC2 effector cytokines, and increases resistance to high-dose infection with *T. muris*^73^. This may be in part due to increased IL-33 receptor (ST2/IL-33R) expression on ILC2 by VIP, amplifying the responsiveness to activation by IL-33^72,74^. A similar, potentiating effect is observed for intestinal ILC3 and VIP binding to VIPR2 promoting the release of IL-22 and the anti-bacterial immune response in the gastrointestinal tract^75^, although one study reported a suppressive effect of VIP on ILC3^76^. In addition to activation of intestinal ILC2, VIP released by pulmonary neurons induces IL-5 release by ILC2 promoting allergic inflammation^74^. This function seems to be amplified by a direct stimulatory role of IL-5 acting on nociceptors, which accelerated the release of VIP. Thus, the release of VIP and its function on ILC2 may act as an important feed-forward loop in type 2 inflammation. In contrast to the stimulating function of NMU and VIP on ILC2, epinephrine (EPI) signaling through the β_2_-adrenergic receptor (β_2_AR) possesses the propensity to repress ILC2 responses^77^. Intestinal ILC2 colocalize with adrenergic neurons and β_2_AR deficiency results in accumulation of IL-13 expressing ILC2, aggravated eosinophilia and a concomitant reduction in worm burden upon infection with *N. brasiliensis*. This putative nervous-immune crosstalk may prevent overactivation of ILC2 and ILC2-mediated pathologies, as ablation of adrenergic signaling augmented airway inflammation induced by intranasal application of IL-33. In contrast, treatment with β_2_AR agonists impaired the production of ILC2-derived effector cytokines ameliorating airway inflammation^77^ (Fig. 2).

## The intricate interplay of helminths and host metabolism

Helminths are multicellular parasites, and thus require a substantial amount of energy for survival, growth, and replication. A possible link to host metabolism was first extrapolated from epidemiological studies in which a negative correlation between obesity, type 2 diabetes and endemic chronic helminth infections suggested a causal connection^78,79^. This assumption received experimental support from Locksley and colleagues showing that infection with *N. brasiliensis* maintained the metabolic health of mice and glucose tolerance through adipose tissue eosinophilia^80^. Later, based on the knowledge that ILC2 were originally discovered in the mesenteric fat, a direct effect of ILC2 and adipose tissue homeostasis was established^81,82^. Non-obese adipose tissue-infiltrating immune cells mainly consist of AAM, eosinophils, T regulatory cells (T_Reg_) and ILC2^80,81,83,84^. This homeostasis is perturbed in high-fat diet (HFD)-induced obesity or obese or diabetic humans, where a loss of ILC2 coincides with increases in ILC1, neutrophils and inflammatory macrophages contributing to an inflammatory tissue environment by accelerating adipose tissue fibrogenesis and impairing glycemic tolerance^21,84,85^. The role of ILC2 in the regulation of host metabolism was further demonstrated by depletion of ILC2 in wild-type or obese Rag-deficient mice, resulting in weight gain and glucose intolerance^86,87^. Furthermore, treatment of obese mice with IL-33 or IL-25 leads to weight loss and increased glucose tolerance, whereas lack of IL-33 results in weight gain and glucose intolerance^82,83,88^, demonstrating the pivotal function of ILC2 and ILC2-activating cytokines in maintaining the metabolic fitness of the organism.

Yet, how ILC2 control host metabolism remains somewhat elusive. One proposed mechanism is that ILC2-derived IL-5, and IL-13 induce AAM and eosinophil accumulation, and that both cell types promote the beiging of white adipocytes^89,90^. Nonetheless, although AAM were initially suggested to directly induce thermogenesis in brown and lipolysis in white adipocytes^91^, this direct involvement of AAM in adipocyte metabolism was lately challenged^89^. In addition, the function of IL-13 in this context is not entirely clear, but lack of IL-13 is associated with weight loss, reduced eosinophils and AAM in adipose tissue^92^. Despite this overwhelming evidence in support of a protective effect of ILC2 preventing metabolic dysfunction, this concept was recently challenged. Rag-Il2rg-deficient mice lacking ILC do not develop obesity and a recent study showed that transfer of intestinal but not adipose ILC2 restored the capacity of these mice to develop HFD-induced obesity^93^. Although these results may have been influenced by homeostatic expansion of ILC2 following transfer into Rag-Il2rg-deficient mice, this clearly shows that more research is necessary to identify the exact modes of action. In this context, a recent study shed some light on potential neuronal control of adipose tissue ILC2. Sympathetic nerves primed adipose mesenchymal cells to produce glial-derived neurotrophic factor (GDNF) and ablation of GDNF signaling led to a significant defect in ILC2 function shaping host metabolism by reducing glucose tolerance and increasing the propensity for HFD-induced obesity^94^. Besides the function of IL-5 and IL-13, eosinophil and ILC2-derived IL-4 directly prompted the proliferation and differentiation of adipocyte precursors into beige adipocytes^95^, characterized by large quantities of mitochondria and expression of uncoupling protein 1 (UCP1)^90,96^. Upregulation of UCP1 inducing thermogenesis and beiging of white adipose tissue appears to be induced by the release of methionine-enkephalin (MetEnk) from ILC2 in response to IL-33. This leads to increased energy consumption, which prevents obesity and metabolic inflammation^83^. Alternatively, IL-33 may also be able to directly induce beiging of white adipose tissue by regulating the appropriate splicing of *Ucp1* mRNA^97^. A direct mode of action is supported by the fact that IL-33 can prevent inflammation of adipose tissue by inducing lipolysis^98^. As both IL-25 and IL-33 modulate host metabolism, their release in the context of helminth infections may be at the center of the observed metabolic alterations. Indeed, *H. polygyrus* has been shown to attenuate obesity via upregulation of uncoupling protein 1 (*Ucp1*) increasing energy expenditure and lipolysis in adipose tissue. These effects were mediated through the induction of AAM^99^ and dependent on norepinephrine^100^. Besides AAM polarization, the onset of obesity has been further associated with increased nematode induced Th2 and T_Reg_ responses in connection to upregulation of *UCP1*, associated with higher energy expenditure^99^. However, the involvement of ILC2 in respect to cellular metabolic homeostasis is still unclear.

## Metabolic control of ILC2 function

**Fig. 3:**
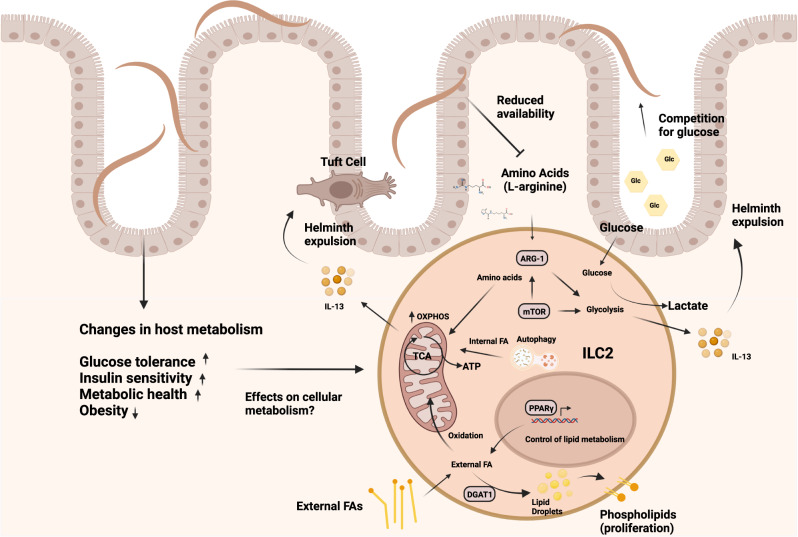
Metabolic regulation of ILC2 in the context of helminth infections. Helminth infections induce several host metabolic changes resulting in increased glucose tolerance, insulin sensitivity and metabolic health, and an overall reduction in obesity. The effects of such changes on cellular metabolism are not known. However, ILC2 can use the acquisition of external FA either for promotion of OXPHOS and ATP generation to fuel anti-helminth immunity via IL-13 or DGAT1-mediated transient storage in lipid droplets and conversation into phospholipids for proliferation in the context of asthma. PPARγ serves as a key regulator of lipid metabolism in ILC2. In contrast, autophagy generates internal FA used in mitochondria, promoting OXPHOS and ILC2 function. AA and glucose metabolism controlled by mTOR represent additional important metabolic pathways, fueling ILC2-mediated immunity. AA are metabolized via OXPHOS, while glucose converted to lactate fuels glycolysis and IL-13 production. The enzyme ARG-1, metabolizing L-arginine, also supports glycolysis. Helminths favor glucose as their primary nutritional source and alter the amino acid availability of the host, which may result in nutrient competition between host and parasites and could potentially impair ILC2-mediated anti-helminth immunity.

As discussed above, besides their direct immune modulatory capacities, helminth infections can alter the metabolism of the host in general and glucose metabolism in particular^101^. Consequently, such metabolic changes may impair the protective immune response against infections and a few studies started to investigate the cellular metabolism of ILC2 in helminth infections. Protective ILC2 responses in helminth infection may circumvent competition with helminths for glucose by metabolizing externally acquired fatty acids (FA) for the generation of energy^101^. Furthermore, malnutrition and vitamin A deficiency is characterized by profound impairment of the adaptive immune system and a selective defect in ILC3^102,103^. Surprisingly, in vitamin A insufficient mice ILC2 cellularity is increased, and IL-13 cytokine production is boosted to sustain barrier integrity and the defense against helminths^102^. In the context of intestinal low-dose *T. muris* infection, ILC2 acquire and utilize fatty acids to boost oxidative phosphorylation to compensate for the loss of the micronutrient vitamin A^101^. Increased mitochondrial oxidation of FA leads to accelerated proliferation and IL-13 production to maintain anti-parasite responses^101^. In addition to externally acquired FA, the mobilization of internal FA through autophagy may be essential to maintain the functionality of ILC2, as deletion of the gene autophagy-related gene 5 (*Atg5*) resulted in accelerated glycolysis but impaired the capacity of ILC2 to oxidize FA in mitochondria^104^. The important implication of this discovery is that ILC2-mediated tissue repair and anti-helminth immunity are maintained in settings of low glucose availability and malnutrition. Since our ancestors, frequently experienced periods of fluctuating nutrient uptake and chronic helminth infections, a switch to fatty acid metabolism represents a host adaptation to ensure barrier integrity in times of severe dietary restriction to extend host survival. As both, malnutrition and helminth infections are largely absent in the Western World, this evolutionary context of malnutrition and competition for nutrients with multicellular parasites may have been overturned by modern lifestyle nowadays, with unknown consequences for ILC-mediated immune responses. In vivo studies have demonstrated that consumption of excess nutrients in form of a high-fat diet (HFD) leads to allergic asthma^105^. Interestingly, in airway inflammation, pathogenic ILC2 require increased uptake of both glucose and lipids, which drive extensive proliferation and pathogenicity^106^. In contrast to the metabolic profile of protective ILC2 in helminth infections^101^, externally acquired fatty acids are used for anabolic processes to build up cellular membranes^106^. Accelerated nutrient acquisition was controlled by IL-33 and resulted in the transient storage of externally acquired FA in lipid droplets to build up cellular membranes, a process controlled by the enzyme diacylglycerol o-acyltransferase 1 (DGAT1). This specific metabolic program is under the transcriptional control of peroxisome proliferator-activated receptor gamma (PPARγ) serving as a key transcription factor controlling the activation and lipid metabolism of ILC2. PPARγ is highly expressed in lung and adipose tissue ILC2^107^ and genetic ablation or pharmacological inhibition of both PPARγ and DGAT1 strongly ameliorated not only ILC2-dependent airway inflammation but also IL-33-mediated colorectal cancer growth^106,108^. Thus, pathogenic activation might rely on the availability of excess nutrients. In support of this idea, ketogenic diets limiting the availability of glucose provide a potent dietary intervention to treat ILC2-driven pathologies such as allergic asthma^106^. Expansion of ILC2 in the airways was dramatically impaired, and although this beneficial effect of the ketogenic diet was independent of the microbiota, other modes of action may be involved. Nonetheless, since ketogenic diets mimic a state of fasting and nutrient deprivation this may explain how periods of malnutrition have counterbalanced the development of chronic inflammation in the past and how the increased incidence of immune pathology in the Western World may be driven by increased consumption of carbohydrate and fat^106^ (Fig. 3).

## How helminths may impact on ILC2 metabolism

Apart from lipid metabolism, ILC2 function is controlled by amino acid (AA) metabolism and glycolysis regulated by high expression of arginase-1 (Arg1) cleaving L-arginine into urea and ornithine^109^. Genetic deletion of *Arg1* led to reduced ILC2 proliferation and total numbers and dampened airway inflammation^109^. Contrary, a study used genetic deletion of *Arg1* specifically in mature ILC2 and found that neither proliferation nor IL-5 or IL-13 cytokine production was affected during helminth infection^110^, suggesting that Arg-1 activity might be depending on the specific inflammatory environment and context. Nonetheless, in confirmation of an important function of AA metabolism, human naïve ILC2 require AA for ATP production via OXPHOS^111^. Upon ILC2 activation biosynthetic demand is higher and branched-chain AA (BCAA) are used to maintain cellular fitness and proliferation^111^, while mTOR controlled glycolysis promotes ILC2 functionality and the production of IL-13^106,111,112^. Helminths may exploit this dependence on AA to avoid expulsion. In fact, infections with *T. muris* increase the amount of AA in the feces^113^, potentially indicating reduced absorption and depletion of AA as a general mechanism of host metabolic manipulation by helminths (Fig. 3). Moreover, at the chronic stage of liver infections with *Opisthorchis felineus* causing hepatobiliary disease, metabolomic analysis of the serum revealed a shift towards lipid metabolism accompanied by depletion of AA^114^. As helminths favor glucose over AA as their primary nutrition source^115,116^, this might represent an adaptation mechanism limiting the activation and expansion of ILC2 in response to helminth infections to prevent expulsion.

Finally, the observed changes in host metabolism could be a direct effect of competition for locally available nutrients, which may also explain why helminths change the composition of the intestinal microbiome in mice and humans^117–123^. Specifically, helminth infections have been associated with increased abundance of carbohydrate-metabolizing *Lactobacillaceae*^121,124–126^. Improved glucose tolerance and preserved insulin resistance in HFD-fed mice by helminths could be mediated by alterations of the intestinal microbiota^78,127^. Consequently, helminth infections may prevent pathogenic activation and chronic inflammation by imposing a metabolic restrain on the host. Some of these aspects have been addressed by probing the beneficial effect of helminth infection in the context of allergies. In confirmation of earlier data^128–130^, infection with *H. polygyrus* protected mice from allergies, an effect abrogated if antibiotics were used to deplete the microbiota^131^. Furthermore, helminth infections elevate the availability of microbial-derived short-chain fatty acids (SCFAs) by increasing the abundance of *Bacteroidetes*^123,132^. This directly results in attenuated allergic airway inflammation by signaling through the cognate SCFA receptor GPR41 (also named free fatty acid receptor 3 (FFAR3))^131^. Cleary, more research is required to validate this concept and to follow up on this understudied but potentially elemental avenue of helminth research.

## Conclusion

Helminths are remarkable in their ability to adapt to their host environment, evading immune activation and expulsion, while being able to feed off and co-exist with their host. Despite having lived with chronic worm infections for centuries, we understand remarkably little about the consequences of untangling such a tight-knit relationship over a relatively short period of time as a consequence of increased hygiene in industrialized countries. A better understanding of this mutualism may be particularly important in regard to the reported alterations in host metabolism implemented by helminths residing at barrier sites. Changes in host metabolism such as increases in glucose tolerance may reflect the need for parasites to gain sufficient access to nutrients, which places intestinal helminths in direct competition with the host and microbiota. Nonetheless, our knowledge of how helminths manipulate host and immune cell metabolism is remarkably limited. Microbiota-derived metabolites produced by intestinal bacteria upon the breakdown of nutrients are emerging as potent activators or inhibitors of cellular function^131,133,134^. Since we currently lack a complete understanding of the mechanism of how helminth infections can influence host metabolism, it is intriguing to speculate that some of the beneficial effects we observe may be directly mediated by such biologically active compounds. Thus, it may be important to focus future research on if or how helminth infections may alter the immune response by regulating the abundance of microbiota-derived metabolites other than SCFA. Given the regulative burden and public health concerns related to using host-adapted parasites therapeutically, unlocking the immune and metabolism-modulating potential of microbiota-derived metabolites may represent a more feasible approach to stall the negative effects associated with a general absence of helminth infections in the Western World.
